# New evidence shows that *Pocillopora* ‘*Pocilloporadamicornis*-like’ corals in Singapore are actually *Pocillopora acuta* (Scleractinia: Pocilloporidae)

**DOI:** 10.3897/BDJ.5.e11407

**Published:** 2017-02-13

**Authors:** Rosa Celia Poquita-Du, Chin Soon Lionel Ng, Jun Bin Loo, Lutfi Afiq-Rosli, Ywee Chieh Tay, Peter A Todd, Loke Ming Chou, Danwei Huang

**Affiliations:** 1 Department of Biological Sciences, National University of Singapore, Singapore, Singapore; 2 Tropical Marine Science Institute, National University of Singapore, Singapore, Singapore; 3 School of Chemical and Life Sciences, Singapore Polytechnic, Singapore, Singapore

**Keywords:** biodiversity, cryptic species, distribution, museum specimens, phylogenetic analysis, *
Pocillopora
*

## Introduction

Numerous hard coral species exhibit substantial intraspecific morphological variation (Foster 1977, Foster 1983, Veron 1995), which is observable at intracolonial, intrapopulation and interpopulation levels (Best et al. 1984). Such variability can arise from genetic differences or phenotypically plastic responses to the surrounding environment (Todd et al. 2004, Pinzón et al. 2013). As coral taxonomy is largely reliant on skeletal morphology (e.g. Veron 2000), such variability can potentially blur species limits and render identification difficult.

The genus *Pocillopora* Lamarck, 1816, is of special interest as it is characterised by exceptionally high levels of phenotypic variation (Veron 2000, Flot and Tillier 2006, Schmidt-Roach et al. 2014a), and its species are frequently used as models in ecological and experimental studies. Due to a lack of clear morphological traits to distinguish among species, some taxa have historically been synonymised. For example, *Pocillopora damicornis* (Linnaeus, 1758) has had at least five synonyms (*P. acuta* Lamarck, 1816; *P. brevicornis* Lamarck, 1816; *P. bulbosa* Ehrenberg, 1834; *P. favosa* Ehrenberg, 1834; and *P. caespitosa* Dana, 1846), which are all considered to be morphological variants of a single complex associated with different environments (Veron and Pichon 1976, Veron 2000, Schmidt-Roach et al. 2013).

Recently, *P. acuta* was re-established as an entirely separate species from *P. damicornis* based on differences in their mitochondrial open reading frame (ORF) sequences (Schmidt-Roach et al. 2014a). *Pocillopora acuta* also differs from *P. damicornis* by possessing more elongated, sharper and thinner branchlets, as well as having dark brown pigmentation around the oral openings of the polyps (Schmidt-Roach et al. 2014a).

The morphological characteristics described for *P. acuta* are exhibited by corals previously identified as *Pocillopora damicornis* in Singapore. Based on this observation, we hypothesise that most of the *Pocillopora* colonies on Singapore’s reefs are likely to be *P. acuta* and not *P. damicornis*. Here, we examine a range of *P.* ‘*damicornis*-like’ (*sensu* Pinzón et al. 2013) colony morphologies, their mitochondrial ORF sequences, and verify their taxonomic identity as the basis for characterisation of *P. acuta* or *P. damicornis* in Singapore.

Results of this study are important for coral diversity records in Singapore and will also help clarify the geographical range limits of morphologically closely-related *Pocillopora* species. As one of the most widespread corals on Singapore's reefs (Huang et al. 2009), these *P.* ‘*damicornis*-like’ colonies are able to settle and grow on natural reefs and even artificial substrates such as seawalls and pontoons (Toh et al. 2017). Consequently, they are widely utilised in reef restoration research (Ng et al. 2016). Their accurate identification, whether they are *P. acuta* or *P. damicornis*, is critical for achieving species diversity targets in local and regional restoration efforts (Mace 2004, Wheeler 2004). Similar to other corals, *Pocillopora* is threatened by habitat loss and climate change, so a clear understanding of species boundaries will facilitate the conservation of species under this genus.

## Materials and methods

**Figure 1.**
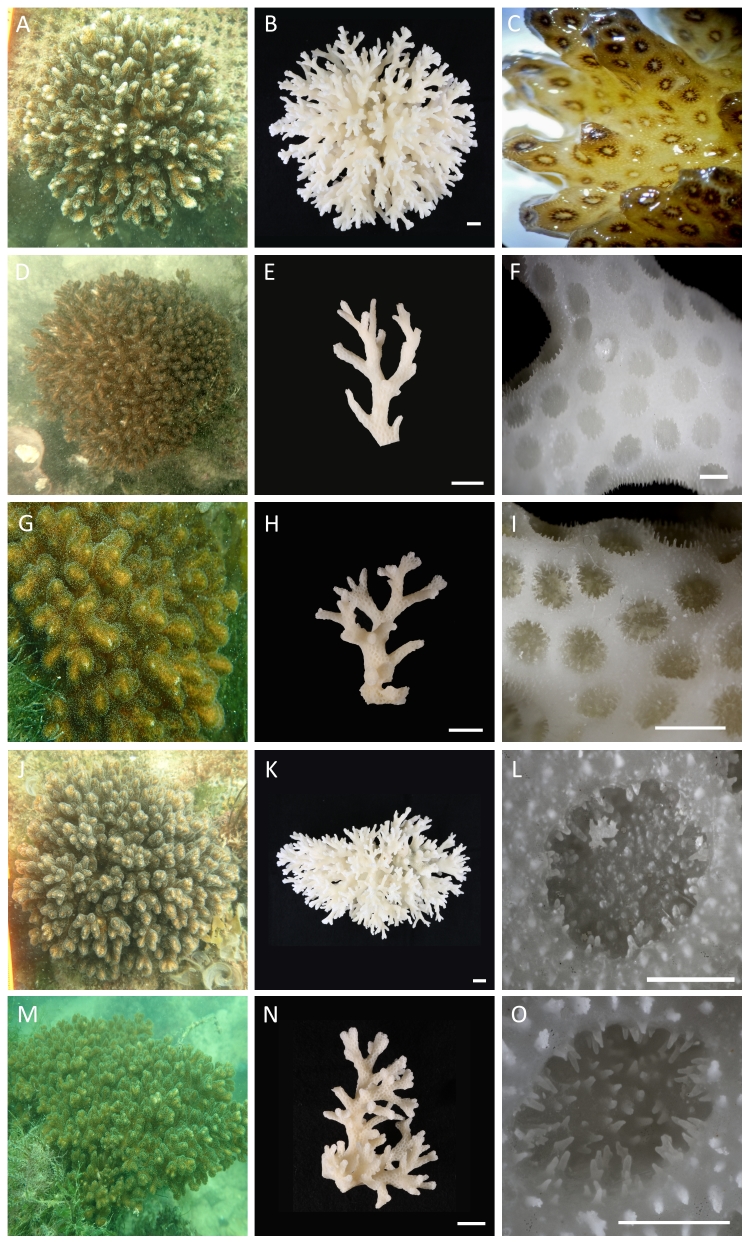
*Pocillopora* specimens examined in this study. *In situ* appearances (A: HD159, D: HD162, G: HD161, J: HD160, M: HD154), with corresponding images of bleached skeletons (B, E, H, K, N). C, live specimen showing brown ring surrounding each oral opening (image by Jenny). F, I, branches from colonies shown in D and G respectively. L, O, calices and septa from colonies shown in J and M respectively. Scale bars represent 1 cm (B, E, H, K, N) and 1 mm (F, I, L, O) respectively.

**Figure 2.**
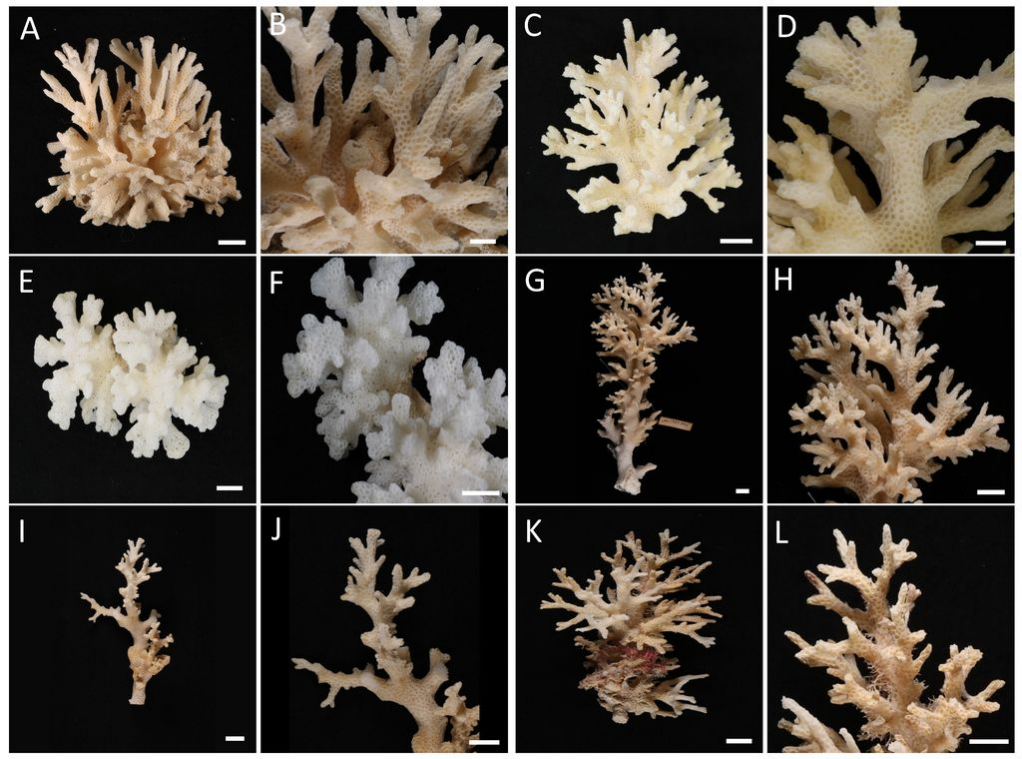
*Pocillopora* specimens previously identified as *P. damicornis* from the Zoological Reference Collection, Lee Kong Chian Natural History Museum, Singapore (A, B: ZRC.1980.20.133; C, D: ZRC.1991.766; E, F: ZRC.1987.1538; G, H: ZRC.1987.1995; I, J: ZRC.1991.763; K, L: ZRC.1987.1537). A–F, colonies with thick branches; G–L, colonies with thinner branches. Scale bars represent 1 cm.

Sixteen *Pocillopora* samples were collected from five coral reef sites across the southern offshore islands of Singapore. We focused on a wide range of colony morphologies that, following Veron and Pichon (1976) and Veron (2000), had been identified previously as *P. damicornis*. We photographed each colony in the field before part or all of it was collected. A 1-cm length of a branch was preserved in 100% ethanol while the rest of the colony sample was soaked in freshwater, bleached in 2% sodium hypochlorite, then rinsed, cleaned and dried. These samples (Fig. 1), along with others loaned from the Zoological Reference Collection (ZRC; Fig. 2) at the Lee Kong Chian Natural History Museum (LKCNHM, Singapore), were photographed and characterised according to the diagnostic characters specified by Schmidt-Roach et al. (2014a).

DNA was extracted by overnight digestion in hexadecyltrimethylammonium bromide (CTAB) and proteinase K, followed by phase separation using phenol: chloroform: isoamyl-alcohol (25:24:1). Polymerase chain reactions primed using FATP6.1 and RORF were performed according to Flot et al. (2008) for amplifying the mitochondrial open reading frame (Flot and Tillier 2007). Reaction products were purified using SureClean Plus (Bioline) and sequenced with the 3730xl DNA Analyzer (Thermo Fisher Scientific). Sequences were deposited in GenBank (accession numbers KY587458–KY587472).

We compiled in Mesquite 3.10 (Maddison and Maddison 2016) the sequences obtained here combined with 146 sequences available in GenBank (Schmidt-Roach et al. 2012, Schmidt-Roach et al. 2013, Marti-Puig et al. 2014), which were derived from *Pocillopora eydouxi* and *P. ligulata*—designated as outgroups in our analyses—as well as *P. damicornis*, *P. acuta*, *P. aliciae*, *P. verrucosa* and *P. meandrina* (Types α, β, δ, γ and m, respectively, according to Schmidt-Roach et al. 2014a). Alignments were carried out in MAFFT 7.205 using default parameters (Katoh and Standley 2013, Katoh and Toh 2008, Katoh et al. 2002), and the 850-bp data matrix was analysed phylogenetically under maximum likelihood and Bayesian optimality criteria.

We used RAxML 8.0.9 (Stamatakis 2006, Stamatakis 2014, Stamatakis et al. 2008) for maximum likelihood analysis, generating 50 alternate runs from distinct parsimony starting trees with the default GTRGAMMA substitution model. Branch supports were assessed via 1000 replicates of bootstrap analysis. For Bayesian analysis, we first selected the most suitable evolutionary model for the data (GTR + I) using jModelTest 2.1.10 (Guindon and Gascuel 2003, Darriba et al. 2012, Posada 2008), following the Akaike information criterion (AIC). Then, Bayesian inference was carried out using MrBayes 3.2.5 (Huelsenbeck and Ronquist 2001, Ronquist and Huelsenbeck 2003, Ronquist et al. 2012), implementing six million generations of Markov chain Monte Carlo in two separate runs and saving a tree every hundredth generation. We used Tracer 1.6 (Rambaut et al. 2014) to assess convergence among the runs, and determined that the first 10001 trees were to be discarded as burn-in.

## Results

**Figure 3.**
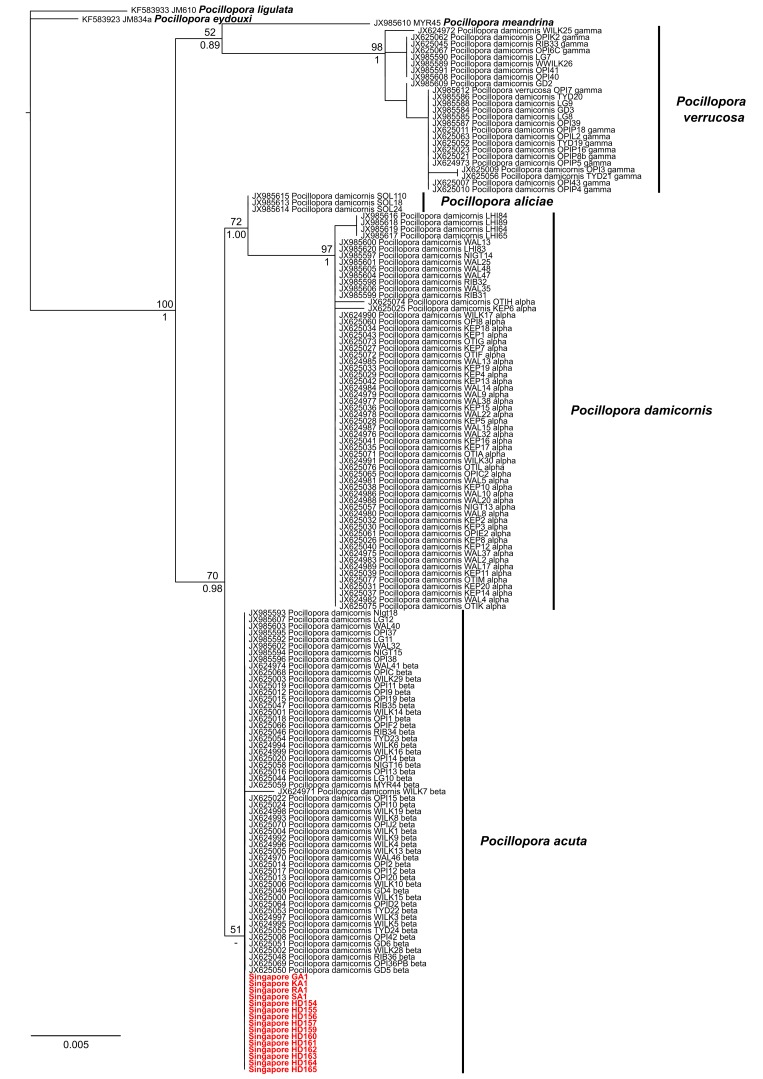
Maximum likelihood tree of seven *Pocillopora* species based on the mitochondrial open reading frame. Colonies from Singapore are shown in red. Bootstrap values (≥ 50) and Bayesian posterior probabilities (≥ 0.85) are shown for supported clades.

Our phylogenetic analysis of seven *Pocillopora* species recovers two moderately-supported monophyletic groups of *P. meandrina* + *P. verrucosa* (*P. damicornis* type γ) and ‘Clade 1’, as defined by Schmidt-Roach et al. (2014a). The latter clade comprises the *P. damicornis* types α, β and δ, which correspond to the newly-delimited taxa *P. damicornis*, *P. acuta* and *P. aliciae*, respectively (Fig. 3).

The topology and statistical supports within this clade match those obtained by Schmidt-Roach et al. (2014a), with only the subclade of type α being highly supported. Types β and δ are only minimally supported by maximum likelihood inference and not supported by Bayesian analysis. Nevertheless, each of these sequence types are supported by nucleotide changes that are fixed within the clade. Type α sequences are distinguished from type β and type δ sequences based on six and four variable sites, respectively, while the latter two types are different at two sites across the 850-bp alignment. Therefore, sequencing errors aside, ORF sequences can help separate the three species effectively despite minimal phylogenetic supports.

### *Pocillopora acuta* Lamarck, 1816

*Pocillopora acuta* Lamarck, 1816, p. 274; Schmidt-Roach et al. 2014a, p. 17; Schmidt-Roach et al. 2014b, p. 11; Kitano et al. 2015, p. 21; Mayfield et al. 2015, p. 1.

*Materials examined*. MNHN-IK-2010-792 (holotype, Muséum national d’Histoire naturelle de Paris, France; type locality: Indian Ocean); see Table 1 for voucher specimens (ZRC, LKCNHM).

*Description*. Colonial, densely caespitose (Fig. 1A, D, G, J, M); branches typically round in cross section, but may become flattened at the tips, which are usually sharply pointed. Branches of colonies in exposed sites thicken and have smaller spacing between branches (Fig. 2A–F), while those in sheltered sites are elongate and slender (Fig. 2G–L). Calices typically oval, with the small diameter ranging between 0.6 and 0.8 mm, and the large diameter between 1.0 and 1.2 mm. Columellae flat. Septa poorly developed, in two equal cycles; 12 septa per corallite (Fig. 1L, O). Coenosteum with fine spinules. Living colony pale-greenish in colour with characteristic darker pigmentation surrounding oral opening of polyps (Fig. 1C).

*Remarks*. Colonies collected from Singapore’s reefs show great variation in branching morphologies, overlapping with those described for *Pocillopora acuta* and *P. damicornis* by Schmidt-Roach et al. (2014a). For instance, *Pocillopora acuta* colonies observed from exposed environments in Singapore have considerably thicker branches (~6 mm) than those shown in Schmidt-Roach et al. (2014a). The pigmentation rings may not be clearly visible for all corallites in every colony; they vary from faint to intense brown. Nevertheless, sequences of the mitochondrial open reading frame from these colonies are all nested within the type-β clade that has been redefined as *P. acuta* (Schmidt-Roach et al. 2014a). The bootstrap support and posterior probability are low, but our sequences exactly match (100% identity) nearly all the published *P. acuta* sequences obtained from Australia and Japan (Fig. 3).

## Discussion

This study contributes to the limited data that have emerged from the South China Sea region on the identity of *Pocillopora* species. The recently-revived species *P. acuta* is described to have a wide distribution reaching from the central Pacific to the Indian Ocean (Schmidt-Roach et al. 2014a). Our results show clearly the presence of *Pocillopora acuta* in Singapore, one of the southernmost localities in the South China Sea. Here, it is found in nearly every offshore reef (Huang et al. 2009), and colonies are common in the shallows (0–4 m below chart datum) but generally do not exist beyond 6 m depth. They thrive in various types of habitats including on artificial substrates such as seawalls and pontoons (Toh et al. 2017), and serve as habitat for diverse ectosymbiont communities (Lee and Sin 2009). The species is hermaphroditic (Kerr et al. 2011) and has been documented to brood monthly in Singapore, just before or after the new moon (Chou and Quek 1993, Toh et al. 2013).

Although we find no contemporary evidence of *P. damicornis* on Singapore’s reefs, the variability of colony branch thickness among specimens from past collections held at the LKCNHM (Fig. 2) appears greater than those collected from the field for this study and overlaps with that of *P. damicornis* as defined by Schmidt-Roach et al. (2014a). Nevertheless, some specimens we sequenced as *P. acuta* here, which were collected from several environments spread across most of the reef localities in Singapore, also had thick branches and rounded tips (e.g. HD154; Fig. 1M, N) diagnostic of *P. damicornis* (Schmidt-Roach et al. 2014a). This wide morphological variation appears to be associated with wave and current exposure, with an increase in branch thickness going from sheltered sites to more exposed ones. It is also possible that the actual *P. damicornis* species had been present before but faced recent losses locally due to pressures from coastal development (Chou 2006, Huang et al. 2009). Hence, a regional investigation with genetic samples from less impacted reefs may help clarify the historical distributions of *P. acuta* and *P. damicornis*.

The taxonomy of South China Sea *Pocillopora* remains poorly understood. A previous study has shown that *Pocillopora* ‘*damicornis*-like’ types 4 and 5 are present in Taiwan, while the Gulf of Thailand only hosts the latter type (Pinzón et al. 2013). More recently, these genetic types 4 and 5 have been delimited as *Pocillopora damicornis* (type α) and *P. acuta* (type β) respectively by Schmidt-Roach et al. (2013), Schmidt-Roach et al. (2014a). These findings were based on the mitochondrial ORF, the same marker we have sequenced here, thus underlining its utility as a reliable method of identifying the *Pocillopora* species present in Singapore.

Broadly, *Pocillopora acuta* is present in both Taiwan and Gulf of Thailand, but *P. damicornis* appears to be limited to the northern South China Sea as it has thus far only been confirmed from Taiwan using the mitochondrial ORF. Further north at the Yaeyama Islands, Japan, *P. damicornis* is present but is likely rare relative to *P. acuta* (Kitano et al. 2015). Despite that, *P. damicornis* is the only *Pocillopora* species detected along mainland Japan to date (Pinzón et al. 2013). All these sequencing work, applied on a wide range of colony and corallite morphologies that overlap with both species (Figs 1, 2), suggests that *P. damicornis* is rare in the South China Sea and southern Japan.

Thus far ranging from the Central Pacific to the Indian Ocean through Singapore, Taiwan and Hawai’i, further sampling may reveal the presence of *P. acuta* in more localities in the Central Indo-Pacific. Overall, the emerging picture shows that most ‘*damicornis*-like’ corals in the southwestern South China Sea region are actually *P. acuta* instead of *P. damicornis*.

## Figures and Tables

**Table 1.** Voucher specimens examined.

**Specimen no.**	**Catalogue no.**	**Locality**	**Latitude, Longitude**	**Date collected**	**Collector**	**Last identification**
RA1	-	Raffles Lighthouse	1.1602°N, 103.7403°E	Oct 2015	R.C. Poquita-Du	*Pocillopora damicornis*
GA1	-	Raffles Lighthouse	1.1602°N, 103.7403°E	Oct 2015	R.C. Poquita-Du	*Pocillopora damicornis*
SA1	-	St. John’s Island	1.2236°N, 103.8452°E	Oct 2015	R.C. Poquita-Du	*Pocillopora damicornis*
HD154	ZRC.CNI.1067	Pulau Subar Darat	1.2158°N, 103.8314°E	Oct 2016	D. Huang	*Pocillopora acuta*
HD155	-	Pulau Subar Darat	1.2158°N, 103.8314°E	Oct 2016	D. Huang	*Pocillopora acuta*
HD156	-	Pulau Subar Darat	1.2158°N, 103.8314°E	Oct 2016	Y.C. Tay	*Pocillopora acuta*
HD157	ZRC.CNI.1068	Pulau Subar Darat	1.2158°N, 103.8314°E	Oct 2016	Y.C. Tay	*Pocillopora acuta*
HD158	-	Pulau Subar Darat	1.2158°N, 103.8314°E	Oct 2016	Y.C. Tay	*Pocillopora acuta*
KA1	-	Kusu Island	1.2257°N, 103.8602°E	Oct 2015	R.C. Poquita-Du	*Pocillopora damicornis*
HD159	ZRC.CNI.1069	Kusu Island	1.2257°N, 103.8602°E	Oct 2016	C.S.L. Ng	*Pocillopora acuta*
HD160	ZRC.CNI.1070	Kusu Island	1.2257°N, 103.8602°E	Oct 2016	C.S.L. Ng	*Pocillopora acuta*
HD161	ZRC.CNI.1071	Pulau Subar Laut	1.2126°N, 103.8334°E	Oct 2016	Y.C. Tay	*Pocillopora acuta*
HD162	ZRC.CNI.1072	Pulau Subar Laut	1.2126°N, 103.8334°E	Oct 2016	Y.C. Tay	*Pocillopora acuta*
HD163	-	Pulau Subar Laut	1.2126°N, 103.8334°E	Oct 2016	Y.C. Tay	*Pocillopora acuta*
HD164	-	Pulau Subar Laut	1.2126°N, 103.8334°E	Oct 2016	Y.C. Tay	*Pocillopora acuta*
HD165	-	Pulau Subar Laut	1.2126°N, 103.8334°E	Oct 2016	Y.C. Tay	*Pocillopora acuta*
-	ZRC.1980.3.20.133	Sentosa	-	Sep 1979	L.T. Chan	*Pocillopora damicornis*
-	ZRC.1987.1537	Pulau Hantu	-	1987	L.M. Chou	*Pocillopora damicornis*
-	ZRC.1987.1538	Pulau Hantu	-	1987	L.M. Chou	*Pocillopora damicornis*
-	ZRC.1991.763	Pulau Hantu	-	1991	Reef Ecology Study Team	*Pocillopora damicornis*
-	ZRC.1991.766	Singapore		1991	Reef Ecology Study Team	*Pocillopora damicornis*
-	ZRC.1987.1995	Singapore	-	-	Reef Ecology Study Team	*Pocillopora damicornis*
